# One-Dimensional Systemic Modeling of Thermal Sensors Based on Miniature Bead-Type Thermistors

**DOI:** 10.3390/s21237866

**Published:** 2021-11-26

**Authors:** Rodolphe Heyd

**Affiliations:** Laboratoire Angevin de Mécanique, Procédés et InnovAtion (LAMPA), Arts et Métiers ParisTech, Boulevard du Ronceray 2, BP 93525, CEDEX 01, F-49035 Angers, France; Rodolphe.HEYD@ensam.eu

**Keywords:** thermal conductivity measurements, miniature NTC thermistor, self-heating methods, systemic modeling, Godunov discretization scheme, SPICE

## Abstract

Accurate measurements of thermal properties is a major concern, for both scientists and the industry. The complexity and diversity of current and future demands (biomedical applications, HVAC, smart buildings, climate change adapted cities, etc.) require making the thermal characterization methods used in laboratory more accessible and portable, by miniaturizing, automating, and connecting them. Designing new materials with innovative thermal properties or studying the thermal properties of biological tissues often require the use of miniaturized and non-invasive sensors, capable of accurately measuring the thermal properties of small quantities of materials. In this context, miniature electro-thermal resistive sensors are particularly well suited, in both material science and biomedical instrumentation, both in vitro and in vivo. This paper presents a one-dimensional (1D) electro-thermal systemic modeling of miniature thermistor bead-type sensors. A Godunov-SPICE discretization scheme is introduced, which allows for very efficient modeling of the entire system (control and signal processing circuits, sensors, and materials to be characterized) in a single workspace. The present modeling is applied to the thermal characterization of different biocompatible liquids (glycerol, water, and glycerol–water mixtures) using a miniature bead-type thermistor. The numerical results are in very good agreement with the experimental ones, demonstrating the relevance of the present modeling. A new quasi-absolute thermal characterization method is then reported and discussed. The multi-physics modeling described in this paper could in the future greatly contribute to the development of new portable instrumental approaches.

## 1. Introduction

The miniaturization of electro-thermal sensors consists of designing and manufacturing ever smaller measuring devices, in order to characterize very limited quantities of material, under a wide variety of operating conditions. This miniaturization has opened up new perspectives in many scientific and technical fields, particularly in medicine, where it has enabled in vivo thermal characterization of biological tissues, that is of crucial importance, for example, in the focal treatment of tumors by magnetic hyperthermia [1].

If some attempts of thermal systems miniaturization at the microscopic scale have been successfully achieved, particularly with the development of labs on a chip, they mainly concern the detection of micro objects [2], thermal imaging, or heat transfer enhancement in micro-fluidics [3]. The precise measurement of the thermal properties of materials is still mainly carried out today at centimeter or millimeter scales [4,5]. Therefore, in this paper, we refer to miniature sensors as sensing elements whose main dimensions are in the order of the millimeter to a few hundred micrometers.

It is clear that the miniaturization of electro-thermal sensors brings undeniable advantages, such as: a lower thermal inertia; a limited invasiveness of the system to be characterized, which is, therefore, less disturbed by the sensor; and the possibility of using small quantities of material, that is highly desirable in nanotechnologies, for example, where the products to be characterized can be very expensive. However, the miniaturization of electro-thermal sensors can also have drawbacks, the most delicate being certainly the problem of the increasing influence of boundary-effects, mainly due to electrical connections. These connections represent unavoidable heat sinks for the sensor, systematically imposing a precise and delicate calibration before any use [6,7]. It is mainly this aspect that makes it very difficult to design new measuring systems using miniature electro-thermal sensors. Under these conditions, it is very desirable to be able to develop systemic modelings, allowing for describing, as accurately as possible, the real operation of thermal sensors in their measurement environment. Thus, a one-dimensional (1D) systemic modeling using a Godunov-SPICE discretization is presented in this paper, in the case of miniature bead -type thermistor sensors with negative temperature coefficient (NTC). This approach allows for a very efficient model of the sensor, its control, and signal processing circuits, as well as the thermal interactions with the material to be characterized, and this within a single workspace (systemic modeling).

There are a large number of thermal characterization methods (depending on the nature of the materials to be characterized), which can be classified mainly into two categories [8,9,10], without being exhaustive:

- the transient methods, among which we can mention: the pulse-decay method [11]; the temperature step methods [6,12,13,14]; the transient hot wire (THW) and the transient hot strip (THS) methods; flash methods; transient plane source methods (TPS); 3ω harmonic methods [15,16];
- the steady-state methods, such as the guarded hot plate and the heated thermocouple methods.

These techniques can be implemented using a wide variety of different thermal sensors (temperature or heat flow), such as: thermocouples, negative temperature coefficient (NTC) thermistors, resistance temperature detectors (RTDs), and radiative sensors.

In the present paper, we will focus on the transient mode use of miniature resistive electro-thermal sensors, for thermal characterization of solid or liquid materials, in the laboratory or in everyday use conditions. These sensors can be classified into two main categories: the hot metal wires (HMW) or hot metal films (HMF) made of pure metal resistors (RTD), such as platinum or nickel; and the negative temperature coefficient (NTC) thermistors, generally composed of semiconducting metal oxides. All these sensors share the same operating principle: the time evolution of their electrical resistance R(t), due to a controlled self-heating, depends on the physical properties (thermal conductivity, density, specific heat, fluid flow) of the material to be characterized, in which the sensor is immersed.

Among these resistive electro-thermal sensors, bead-type thermistors have the advantages of spherical or quasi-spherical symmetry, being non-traversing and small in size, unlike wire sensors (which are traversing or large in size) and hot film sensors (which are usually non-symmetrical composite structures [17]). In contrast, RTDs have the great advantage of obeying a law of variation of the resistance as a function of temperature which is linear with a very good approximation, while NTCs obey a non-linear law of variation. The linearity property of RTDs greatly simplifies the development of signal processing circuits and allows a wide variety of applications, ranging from thermal characterization of materials (THW method, 3ω method) to flow measurement (hot wire or hot film anemometry).

In contrast, the non-linearity of NTCs has certainly been a major obstacle to the development of various thermal characterization methods using them. If NTCs are indeed used for transient thermal characterization of pulse-decay or temperature step types, however, we do not find in the literature any use in harmonic method of 3ω type for example. Thus, main objective of this paper is to propose an accurate 1D systemic multi-physics modeling of bead-type thermistors, which are non-linear but very easy to implement sensors. This systemic modeling should, on the one hand, help in the development of new thermal characterization methods using miniature bead-type NTCs; on the other hand, it should help to better understand the operating mode of existing methods.

The implementation modes of miniature resistive sensors used in material characterization can be classified into two main categories:

- the source/sensor(s) mode [18,19,20], which implements separate heat source and sensors. This operating mode requires an accurate and reproducible positioning of the sensor(s) relative to the heat source; and
- the self-heating mode [10], in which the sensitive element serves as both a heat source and a temperature sensor. The basic principle of this mode of operation is that the less heat conducting the surrounding materials to be characterized, the greater the self-heating of the sensor element. Thus, the average temperature $T_{c}\left(t\right)$ of the sensor and the electrical power $P_{e}\left(t\right)$ it dissipates are, in most situations, the basic informative signals considered in electro-thermal methods used for thermal characterization.

While the 1D systemic modeling proposed in this work allows the simulation of both characterization modes, it is the self-heating mode that will be mainly exposed here.

Simulation has become an essential tool in the development of electronic circuits. In electronics, the SPICE (Simulation Program with Integrated Circuit Emphasis) code, developed at Berkeley in the early 1970s [21], has been widely adopted and has indeed become a true industrial standard. While SPICE excels at solving the ordinary differential equations (ODEs) that describe the temporal operation of electrical circuits, it lacks the intrinsic ability to solve the partial differential equations (PDEs) that describe heat transfers and spatiotemporal evolutions of the temperature within the sensor and the material being characterized. To correct this deficiency, it is necessary to develop accurate models of electro-thermal sensors, which integrate interactions between thermal quantities and electrical signals. In reference [22], Heyd et al. used the capability of SPICE for fast and accurate Laplace transform inversion to solve the unsteady heat equation in a hot wire heated by Joule effect and submitted to a fluid flow (hot wire anemometer). In this context, time and position were treated as continuous variables, allowing almost unlimited accuracy in the simulation of the different modes of operation of hot-wire anemometers. Other approaches are possible [23,24,25,26,27,28,29], which generally implement, on the one hand, a spatial discretization of the medium to be characterized (and possibly also of the sensor) and uses, on the other hand, an analogy to transform this discretization into an analogous electrical circuit. This analogous circuit is then coupled to the electrical control signals.

A hybrid approach is used in the present work. The material to be characterized and the composite bead-type NTC (core and sheath) are all discretized in spherical symmetry (1D equivalent model) using a Godunov-type numerical scheme [30], while the electrical connection wires of the thermistor are modeled using thermal resistors. The results obtained using this numerical model in SPICE are then compared with the experimental results obtained with pure glycerol at rest. Then, different effective parameters of the 1D equivalent model are adjusted to allow the best possible reproduction of the experimental measurements.

## 2. Materials and Circuits

### 2.1. Glycerol

In order to avoid natural convection as much as possible during the development of the 1D systemic NTC model, glycerol, a non-ionic liquid with high viscosity, was chosen as test fluid.

Because of the high hygroscopicity of glycerol, only fresh liquid (Sigma-Aldrich, St, Saint-Quentin-Fallavier, France) was used, and sealing precautions were taken to avoid dissolution of atmospheric water vapor into the liquid during measurement and storage.

### 2.2. NTC Bead-Type Thermistor

Accurate and reproducible determination of the thermal properties of materials using a miniature NTC bead-type thermistor requires a good understanding of the different heat exchanges that take place within the system. It is difficult, if not impossible, to consider absolute measurements of thermal conductivity and diffusivity using NTC thermistors, without a precise knowledge of their internal structure and of the influence of electrical contacts.

#### 2.2.1. Composition—Electrical Properties

**Figure 1:**
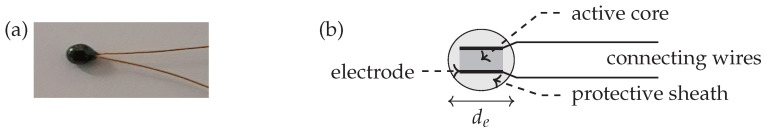
(**a**) Precision miniature epoxy encapsulated bead-type 44004 NTC thermistor (2252@25) from Omega and (**b**) its schematic composition. The length of the connecting copper wires is equal to L=76 mm in the present case.

The sensor used in this work is a miniature high precision epoxy encapsulated bead-type thermistor of type 44004 from Omega, whose resistance at a temperature of 25 °C is equal to 2252 Ohms (2252@25; see Figure 1a). This type of high precision thermistor was chosen both for its quasi-spherical shape and small size and, on the other hand, for its interchangeability. To avoid electrical conduction problems in ionic solvents, such as water, a thin layer of insulating varnish has been applied to the NTCs copper connecting wires.

Miniature NTC thermistors of bead-type generally consist of an active core (see Figure 1), composed of a semiconducting metal oxide with negative temperature coefficient, protected by a glass, nylon or epoxy sheath. While the active core can sometimes have a spherical shape, it is, rather, usually in the shape of a slab (see Figure 1b). In order to allow the circulation of an i(t) electric excitation current through the core, two small metal electrodes are deposited on it, also allowing for measurement of its electric resistance R(T), which depends on the core average temperature *T*.

The external shape of the NTC is usually a prolate spheroid, whose characteristic dimension $d_{e}$ is of the order of the millimeter or less (2.4 mm in the case of 44004 model). Two small diameter copper wires (in the order of a few hundred micrometers: 180μm for 44004) ensure the electrical connection of the active core with the control and processing circuit of the electro-thermal signal.

To measure the temperature *T* of the NTC active core, it is necessary to be able to relate *T* to the value of the NTC electrical resistance R(T). There are several common laws used to describe the variations in the electrical resistance *R* (in Ω) of the active core as a function of its equilibrium temperature *T* (in K). One of the most used is certainly the law of Steinhart and Hart [31]:

(1) $$\frac{1}{T}=c_{0}+c_{1}\ln\left(R\right)+c_{3}\ln{\left(R\right)}^{3},$$

where the constants $c_{0}$, $c_{1}$, and $c_{3}$ are obtained by a precise calibration (at least three points) of the thermistor considered, in the temperature range visited experimentally. In the case of thermistor 2252@25, for example, the values of R(T) (taken from the “Thermistor Resistance vs. Temperature” table provided by Omega) in the range $\left[-80,120\right]{\;}^{\circ }C$ allow the following coefficients to be found (in K${}^{-1}$): $c_{0}=1.47\times 10^{-3}$, $c_{1}=2.38\times 10^{-4}$, and $c_{3}=1.04\times 10^{-7}$.

Simpler models can also be used [32], with only two coefficients. For example:

(2) $$R\left(T\right)=\exp\left(\frac{1}{b_{1}T}-\frac{b_{0}}{b_{1}}\right)=R\left(T_{\mathrm{ref}}\right)\exp\left[\beta \left(\frac{1}{T}-\frac{1}{T_{\mathrm{ref}}}\right)\right],$$

where $T_{\mathrm{ref}}$ is typically chosen equal to 298.15 K. In the case of the 2252@25 thermistor, we have obtained $R_{\mathrm{ref}}=R\left(T_{\mathrm{ref}}\right)=2252\;\Omega$ and β=3864.5 K. This kind of two-factor relationship being less precise than (1); therefore, it is recommended to experimentally determine the coefficients $\left(b_{0},b_{1}\right)$ or $\left(R_{\mathrm{ref}},\beta \right)$ in the range of temperatures investigated during the thermal characterization.

We now review the main energy transfers that take place within the thermistor and its surrounding environment. During a typical liquid thermal characterization experiment, the average electrical power $P_{e}$ dissipated by the thermistor is in the range of 1 to 10 mW. The corresponding temperature variations ΔT involved are then in the order of a few Kelvins, thus generally being much lower than the working temperatures.

Considering the 2252@25 Omega thermistor used in this work, having an average external radius $a_{e}\approx 1.2$ mm, typically traversed during Δt=30 s by an electric current $I_{e}=2$ mA, this corresponds to an average dissipated electric power $P_{e}\approx 10$ mW. When this thermistor is immersed in pure glycerol (at rest), at the working temperature $T_{0}=294$ K, the maximum temperature variations in the active core are about $\Delta T_{c}=T_{c}-T_{0}\approx 1.3$ K and $\Delta T_{e}=T_{e}-T_{0}\approx 0.8$ K for the outer surface of the protective sheath. Considering these orders of magnitude typical of a thermal characterization experiment, we can now analyze the main sources of thermal losses and poor thermal contacts:

- Radiative heat losses $P_{r}$ at the external surface of the NTC: These losses can be estimated using Stefan–Boltzmann’s law: $P_{r}=\sigma \varepsilon \Sigma_{e}\left(T_{e}^{4}-T_{0}^{4}\right)\approx 4\sigma \varepsilon \Sigma_{e}T_{0}^{3}\left(T_{e}-T_{0}\right)$. Under the working conditions presented above, and considering a maximal emissivity ε=1, we obtain $P_{r}\approx 0.1$ mW. Therefore, it is justified to neglect $P_{r}$ in front of $P_{e}$. Under the typical working conditions described in this work, it can be assumed that radiation losses have a negligible influence on the thermal characterization of materials using a miniature bead-type thermistor.
- Thermal losses $P_{L}$ at the electrical contacts between the thermistor and the control circuit: It is difficult to precisely estimate these heat losses because they are closely linked to the nature of the experimental system implemented: electrical insulation of the connecting wires (using a varnish or silicone) and length *L* of the connecting wires; use of metal or plastic mounting probe.
  Under the operating conditions described above, and for varnish insulated copper wires, with a diameter $d_{w}=0.18$ mm and a length of L=1 cm, immersed in pure glycerol at rest, a finite element modeling has led to heat losses typically in the order of 3 mW.
  Therefore, heat losses via the electrical connections are considerable and can in no way be neglected in the modeling of the thermistor.
- Convective losses $P_{c}$ when the material to characterize is a fluid: Convective losses always lead to an additional heat extraction from the sensor and are at the origin of an overestimation in the measurement of the conductivity of the fluid to be characterized. Therefore, it is essential to limit these convective losses if we want to correctly estimate the thermal conductivity of the fluid to be characterized. This requirement dictated the choice of glycerol as a test liquid for 1D systemic modeling of NTCs. The thermal power evacuated from the thermistor to the fluid can be evaluated in the presence of natural convection, from the Churchill correlation [33], which gives the following Nusselt number expression, valid for natural convection around a sphere:
  (3) $$\mathrm{Nu}=2+\frac{0.589\;{\mathrm{Ra}}_{d_{e}}^{1/4}}{{\left[1+{\left(0.469/\mathrm{Pr}\right)}^{9/16}\right]}^{4/9}},$$
  where Pr=ν/α>0.5 is the Prandtl number of the fluid, and ${\mathrm{Ra}}_{d_{e}}=g\beta d_{e}^{3}\Delta T_{e}/\nu \alpha <10^{11}$ is the Rayleigh number for the bead-type thermistor, with: $g=9.81\;m\cdot s^{-2}$ the acceleration of gravity; ν=η/ρ the kinematic viscosity of the fluid $\left(m^{2}/s\right)$; α=k/ρc its thermal diffusivity $\left(m^{2}/s\right)$; and β its volumetric expansion coefficient $\left(K^{-1}\right)$. Note that, in the absence of natural convection (${\mathrm{Ra}}_{d_{e}}=0$), the Nusselt number takes the limiting value Nu=2, which corresponds to pure thermal conduction through the liquid at rest. Typical values (at $20{\;}^{\circ }C$) of water physical properties are collected in Table 1. Typical values of glycerol properties at 20 ${}^{\circ }C$ can be found for example in [34].
  Using the values of Table 1 for $\Delta T_{e}=0.8\;K$, we find in the case of glycerol that Nu≈2.5, which remains close to the value obtained in the absence of natural convection, and $P_{c}=0.8\;\mathrm{mW}$, which is negligible compared to $P_{e}$. It can be concluded that it is acceptable, for temperature variations $\Delta T_{e}⩽1.0\;K$ near room temperature, to neglect the contribution of natural convection around the NTC in the case of immersion in glycerol.
  In contrast, in the case of NTC immersion in water, considering a temperature difference $\Delta T_{e}=0.5\;K$ (thermistor heating is less here than in the case of glycerol) near room temperature, we find that Nu≈3.7, which is quite different from the values obtained in the absence of natural convection, and $P_{c}=3.8\;\mathrm{mW}$, which can no longer be ignored.
- Poor thermal contacts: There are two main sources of poor thermal contact here: the thermal contacts between the active core of the NTC and its protective sheath and between the sheath and the surrounding medium to be characterized. If the latter is a fluid (which is the case in the present study), we can assume that the corresponding sheath/fluid thermal contact resistance is negligible. In contrast, the contact resistance $R_{c}$ between the sheath and the core is potentially significant, unknown and a priori different from one sensor to another and will be taken into account in the 1D systemic modeling proposed in this study.

#### 2.2.2. Heat Transfer through NTC and Surrounding Medium

Despite its small size, a miniature bead-type NTC is a complicated object from a heat transfer point of view. Indeed, the presence of both metallic electrodes and connecting wires, but also of an active core that can be slab-shaped and surrounded by a prolate spheroid protective shell, does not strictly allow for attribution of particular simplifying symmetries to the T(M,t) system temperature field. As a result, an exact description of the thermal behavior of the “Fluid+ NTC + control circuits” multi-physics system would require a three-dimensional (3D) modeling of heat transfers within the system. Unfortunately, the complexity of such a 3D finite element modeling is a significant obstacle to the development of an efficient and fast systemic modeling of NTCs used in thermal characterization.

**Figure 2:**
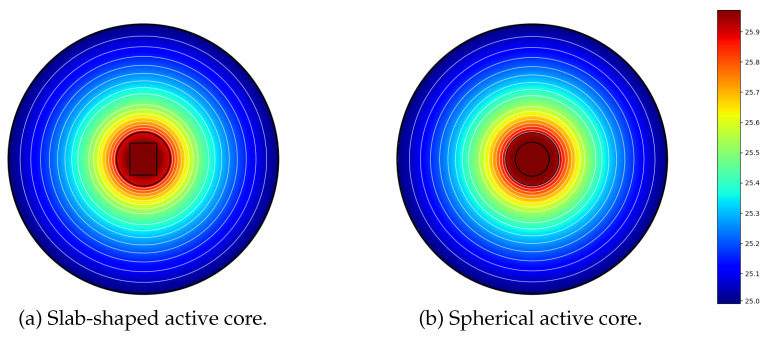
Finite elements modeling of heat transfers through the multi-physics system: NTC + Fluid, with: (**a**) a slab-shaped active-core; (**b**) a spherical active core. In both cases, the same materials were considered for the sheath (epoxy, external radius $a_{s}=1$ mm), the active core (semiconductor material), and for the surrounding fluid (radius $a_{f}=5$ mm) to be characterized.

However, and given the small size of the miniature bead-type thermistor relative to the volume of fluid to be characterized, the heat transfers outside the active core depend very little on its shape (sphere or slab), as illustrated by the finite element modeling results presented in Figure 2. These results were obtained by using the same constant and uniform heating power density ${\dot{q}}_{e}$ in both cases. The effective radius $a_{c}$ of the equivalent spherical active core (see Figure 2b) was adjusted here to obtain the same core temperature as in the slab-shaped core case (see Figure 2a). Note that, in this case, the volume $V_{c}=\frac{4}{3}\pi a_{c}^{3}$ of the equivalent sphere is equal to the volume of the slab.

Based on the results presented in Figure 2, it can be concluded that, regardless of the shape (sphere or slab) of the NTC active core, it is possible to propose an approximate 1D modeling of a miniature bead-type NTC, which uses an equivalent spherical active core of effective radius $a_{c}$. This simplification allows us to propose a quasi-1D modeling of the NTC, with spherical symmetry. Note that, in the case of spherical core NTCs, the effective radius $a_{c}$ can be identified with the exact radius of the active core.

#### 2.2.3. Mathematical Modeling

An elementary mathematical model, that is frequently used to describe the thermal behavior of a miniature NTC of bead-type, consists of assimilating the whole composite sensor to a single sphere of temperature $T_{c}\left(r,t\right)$, with an effective radius *a*, and constant effective thermal properties: *k* (thermal conductivity) and α (thermal diffusivity) [12]. The self-heating of the sensor by the Joule effect is usually modeled by a uniform power density ${\dot{q}}_{e}=P_{e}\left(t\right)/\frac{4}{3}\pi a_{c}^{3}$, where $P_{e}\left(t\right)$ is the electrical power supplied by the driving circuit to the active core of the NTC. The heat transfers through the NTC thermistor, being solely of a diffusive nature, and its temperature $T_{c}\left(r,t\right)$, thus, obeys the following heat equation in spherical symmetry [36], with *r* being the distance to the center of the sphere:

(4) $$\frac{1}{r^{2}}\frac{\partial }{\partial r}\left[r^{2}\frac{\partial }{\partial r}T_{c}\left(r,t\right)\right]+\frac{{\dot{q}}_{e}\left(t\right)}{k}=\frac{1}{\alpha }\frac{\partial }{\partial t}T_{c}\left(r,t\right)\;\;\;\;\;\;\;0\leq r\leq a,$$

with $T_{c}\left(0,t\right)$ finite and $T_{c}\left(r,0\right)=T_{0}$.

**Figure 3:**
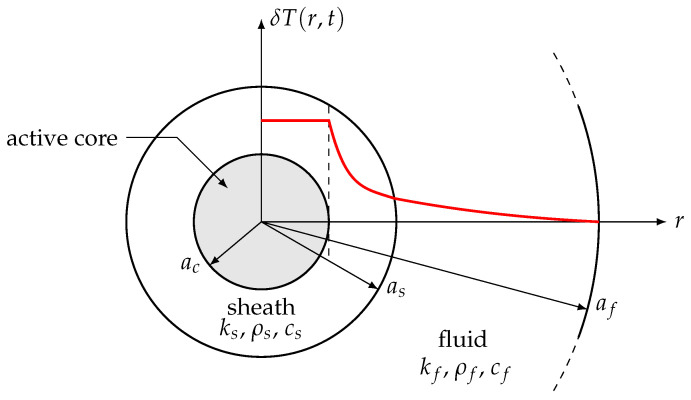
Ideal 1D effective model of a miniature bead-type NTC, immersed in a fluid at rest (with thermal conductivity $k_{f}$, density $\rho_{f}$, and specific heat $c_{f}$). The red curve represents effective variations of the $\delta T=T\left(r,t\right)-T_{0}$ temperature through the system, as a function of *r*, at a given time *t*.

According to the results and discussions of Section 2.2.2, it is also considered in the present work that the thermistor is spherical in shape, of radius $a_{s}$, but it is decomposed into two concentric parts (see Figure 3): the active semiconducting core (Figure 3c) of effective radius $a_{c}<a_{s}$, thermal conductivity $k_{c}$, and temperature $T_{c}\left(r,t\right)$; the insulating protective sheath (s) of effective thickness $e_{s}=a_{s}-a_{c}$, thermal conductivity $k_{s}\ll k_{c}$, density $\rho_{s}$, specific heat $c_{s}$, and temperature $T_{s}\left(r,t\right)$. As in the case of the model proposed by Balasubramaniam et al. [12], the present ideal model does not take into account at this stage the losses due to the connecting wires. These defects will be modeled later using appropriate thermal resistances.

Due to the usual high value of the thermal contrast (or thermal ratio $k_{c}/k_{s}$) between the core and the sheath of the NTC, the temperature $T_{c}\left(r,t\right)=T_{0}+\delta T_{c}\left(r,t\right)$ of the high thermal conductivity core can be considered as uniform to a very good approximation (see Figure 2). Numerical simulations made with the multi-physics finite element solver FlexPDE have shown that, in all the cases under study here, the spatial variations in the temperature $\delta T_{c}\left(r,t\right)$ of the core were absolutely negligible: $1-|\delta T_{c}\left(0,t\right)/\delta T_{c}\left(a_{c},t\right)|<0.1\%$. Thus, it is possible to consider here that the temperature $\delta T_{c}$ only depends on the time *t*. Therefore, the energy balance of the highly conductive core can be written, using the spherical symmetry of the equivalent 1D model, as:

(5) $$\frac{4}{3}\pi a_{c}^{3}\rho_{c}c_{c}\frac{dT_{c}}{dt}=\frac{4}{3}\pi a_{c}^{3}{\dot{q}}_{e}+4\pi a_{c}^{2}k_{s}{\left(\frac{\partial T_{s}}{\partial r}\right)}_{r=a_{c}},$$

where $\rho_{c}$ and $c_{c}$ are the density and the specific heat of the active core, respectively, and $T_{s}$ is the temperature of the sheath, at the interface with the active core at $r=a_{c}$.

On the other hand, the temperature $T_{s}\left(r,t\right)$ of the sheath obeys a diffusive heat equation without source term, which is written, still using the spherical symmetry of the equivalent 1D model, as:

(6) $$\frac{1}{r^{2}}\frac{\partial }{\partial r}\left[r^{2}\frac{\partial }{\partial r}T_{s}\left(r,t\right)\right]=\frac{1}{\alpha_{s}}\frac{\partial }{\partial t}T_{s}\left(r,t\right)\;\;\;\;\;\;\;a_{c}<r<a_{s},$$

where the thermal diffusivity $\alpha_{s}$ of the sheath is supposed to be constant. The thermal contact between the solid sheath (s) and the solid core (c) being a priori not perfect, we can only assume here the continuity of the heat flow at the interface $r=a_{c}$ between (c) and (s):

(7) $$k_{c}{\left(\frac{\partial T_{c}}{\partial r}\right)}_{r=a_{c}}=k_{s}{\left(\frac{\partial T_{s}}{\partial r}\right)}_{r=a_{c}}.$$

The surrounding material (f) to be characterized is assumed to be homogeneous, at rest, of semi-infinite extent and constant thermal conductivity $k_{f}$ and diffusivity $\alpha_{f}$. Two situations are often considered, depending on the physical nature of the surrounding material, delimited by the radius $r=a_{f}$:

- (f) is an inert (non-biological) material at rest, with no heat source term. In this case, its temperature $T_{f}\left(r,t\right)$ also obeys a diffuse heat transfer equation, with spherical symmetry:
  (8) $$\frac{1}{r^{2}}\frac{\partial }{\partial r}\left[r^{2}\frac{\partial }{\partial r}T_{f}\left(r,t\right)\right]=\frac{1}{\alpha_{f}}\frac{\partial }{\partial t}T_{f}\left(r,t\right)\;\;\;\;\;\;\;a_{s}<r<a_{f},$$
- (f) is a biological material, which obeys a differential equation of the Penne type (bioheat transfer equation), for example [37,38]:
  (9) $$\frac{1}{r^{2}}\frac{\partial }{\partial r}\left[r^{2}\frac{\partial }{\partial r}T_{f}\left(r,t\right)\right]+\frac{{\dot{q}}_{f}}{k_{f}}+\frac{\omega \rho_{b}c_{b}}{k_{f}}\left(T_{0}-T_{f}\right)=\frac{1}{\alpha_{f}}\frac{\partial }{\partial t}T_{f}\left(r,t\right)\;\;\;\;\;\;\;a_{s}<r<a_{f},$$
  where ${\dot{q}}_{f}$ and $\omega \rho_{b}c_{b}\left(T_{0}-T_{f}\right)$ are the metabolic and the perfusion heat source terms, respectively. The perfusion term describes the heat transfer between the medium (f) (biological tissues) and blood (b) flowing through the veins and arteries, with a volumetric mass flow $\rho_{b}\omega$ (kg·m${}^{-3}\cdot$s${}^{-1}$) and blood specific heat $c_{b}$.
  The initial condition to be considered for the partial differential Equation (8) or (9) is $T_{f}\left(r,0\right)=T_{0}$. The boundary condition to consider at $r=a_{f}$ depends on the thermal characterization apparatus that is used. We suppose here that the medium to characterize is in perfect thermal contact at $r=a_{f}$ with a thermostat at the temperature $T_{0}$:
  (10) $$T_{f}\left(a_{f},t\right)=T_{0}.$$
  While both situations (8) and (9) can be studied in the same way with the systemic modeling presented in this work, it is the (8) case that will be presented here in detail, both from a numerical and an experimental point of view.
  Finally, a perfect thermal contact is assumed between the thermistor sheath and the medium to be characterized (fluid at $r=a_{s}$), the following continuity relationships must then be satisfied at all times:
  (11) $$T_{s}\left(a_{s},t\right)=T_{f}\left(a_{s},t\right)\;\;\;\;\;\;\mathrm{and}\;\;\;\;\;\;k_{s}{\left(\frac{\partial T_{s}}{\partial r}\right)}_{r=a_{s}}=k_{f}{\left(\frac{\partial T_{f}}{\partial r}\right)}_{r=a_{s}}.$$

The simultaneous solving of the (PDEs) partial differential Equations (5), (6) and (8) (or (9)), together with the boundary conditions (7), (10), (11), and the initial condition $T_{c}\left(0\right)=T_{s}\left(0,t\right)=T_{f}\left(0,t\right)=T_{0}$, allows for determination of the temporal evolution of the core temperature $T_{c}\left(t\right)$, which depends both on the medium to be characterized and the electrical power $P_{e}\left(t\right)$ used to excite the thermistor.

Thus, the 1D systemic approach presented in this work consists of solving the PDEs of the equivalent system, while, at the same time, taking into account the operation of the electronic circuits used to provide the power $P_{e}\left(t\right)$ to the thermistor.

The electronic circuit that was used for both the experimental measurements and the systemic modeling is described in detail in the following paragraph.

### 2.3. Electronic Circuit

**Figure 4:**
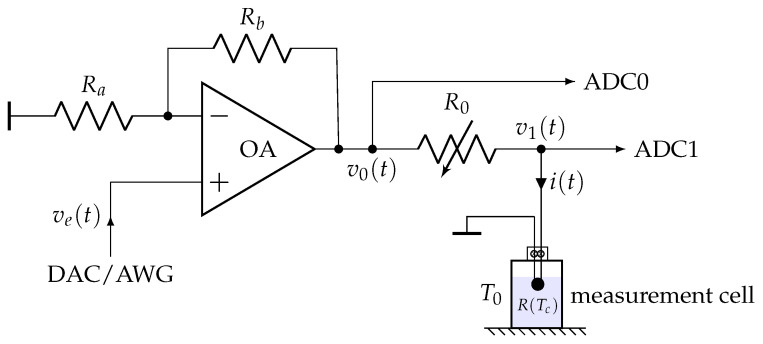
Amplified divider bridge circuit used both to control the self-heating of the NTC bead-type thermistor and measure the time variations of its electrical resistance $R(T_{c})$. This circuit can be used in both transient and frequency modes. ADC0 and ADC1 are two inputs of an Analog to Digital Converter (ADC). The circuit can be excited either by using a Digital To Analog Converter (DAC) or by using an Arbitrary Waveforms Generator (AWG).

The electronic circuit shown in Figure 4 was used for both experimental measurements and systemic modeling. This is a typical example of an amplified voltage divider bridge circuit, quite versatile, which can be adapted according to the implemented resistive sensor (NTC or RTD) and can be used both in transient and frequency modes.

A Measurement Computing (MC) USB-2537 multi-functions data acquisition board has been used for measurements, providing one 16 bits DAC output and two 16 bits ADC inputs (ADC0 and ADC1). The following precautions must generally be observed when implementing transient thermal characterization methods with the circuit shown in Figure 4:

- Since the temperature variations due to self-heating are generally of small amplitude (a few Kelvins at most), it is necessary to use an analog-to-digital converter (ADC) with a resolution of at least 14 bits. Moreover, as thermal phenomena are generally relatively slow (with time constants typically in the order of a few tenths of a second or more), a sampling period of around 10 ms or more is, therefore, sufficient in most cases. A digital-to-analog converter (DAC) with a resolution of 12 bits or better can be used to provide the excitation signal $v_{e}\left(t\right)$ for transient methods. If the sensing element is to be excited in current rather than voltage, a voltage-to-current converter (of the Howland source type) can be used instead of the non-inverting amplifier shown in Figure 4.
- The inputs ADC0 and ADC1 must have a sufficiently large input impedance $Z_{e}$ (typically, $Z_{e}>10^{5}\;\Omega$. In the case of USB-2537: $Z_{e}=10^{7}\;\Omega$) to not load the circuit. If necessary, a high impedance buffer (voltage follower) can be used to isolate the control circuit from the influence of the ADC.
- The operational amplifiers (OA) used in circuit Figure 4 must operate in the linear regime. If the self-heating of the sensor requires an electric current with an intensity i(t)>10 mA, then, the use of an operational amplifier capable of delivering high currents should be considered. This could be the case, for example, with low-resistance platinum wires, whose value is close to 1Ω. In this case, a typical average electrical power $P_{e}=10$ mW requires about 100 mA electrical excitation current.
  To make the set-up as versatile as possible, a power OA of type L272 (delivering currents up to 1 A without significant harmonic distortion) was systematically used.
- The working (or baseline) temperature $T_{0}$ must be precisely regulated, usually by means of a temperature controlled bath. The variations $\Delta T_{0}$ of the working temperature must be negligible in front of the maximal temporal variations of the sensor core temperature $\delta T_{c}\left(t\right)$, due to self-heating. An accuracy of $\Delta T_{0}\approx 0.01\;K$ is sufficient in most cases.

## 3. 1D Systemic Modeling

As mentioned before, an approximate 1D systemic modeling of a bead-type NTC is considered in this work, which consists essentially of assuming that heat transfers through the NTC present the spherical symmetry. This 1D systemic modeling of a bead-type NTC relies first on a suitable spatial discretization of the heat Equation (6) in the sheath and (8) or (9) in the medium to be characterized.

The discretization process of the present thermal problem, as well as different elementary applications of this discretization, is introduced in the present paragraph.

### 3.1. General Approach

**Figure 5:**
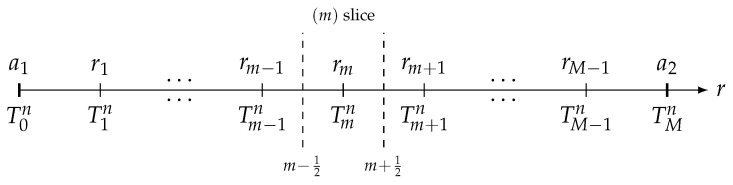
Spatial discretization of the spherical shell $\left(\Omega \right)=\left(a_{1},a_{2}\right)$ into *M* slices of equal thickness $\delta_{r}=\left(a_{2}-a_{1}\right)/M$.

In all cases considered in the present study, we are dealing with (equivalent) domains having the shapes of spherical shells, for which the discretization schemes are, therefore, perfectly similar. Therefore, we choose to discretize the partial differential Equation (9), which has the most general expression here. This PDE is then rewritten in the following general self-explanatory form:

(12) $$\frac{1}{\alpha }\frac{\partial }{\partial t}T\left(r,t\right)=\frac{1}{r^{2}}\frac{\partial }{\partial r}\left[r^{2}\frac{\partial }{\partial r}T\left(r,t\right)\right]+\frac{\dot{q}}{k}+\frac{\omega \rho_{b}c_{b}}{k}\left(T_{0}-T\right)\;\;\;\;\;\;\;a_{1}<r<a_{2},$$

where α=k/ρc is the thermal diffusivity of the physical domain $\left(\Omega \right)=\left(a_{1},a_{2}\right)$. The PDE (12) can be rewritten in the following form:

(13) $$\rho c\frac{\partial }{\partial t}T\left(r,t\right)=k\frac{\partial^{2}}{\partial r^{2}}T\left(r,t\right)+\frac{2k}{r}\frac{\partial }{\partial r}T\left(r,t\right)-h_{\omega }\left(T-T_{0}\right)+\dot{q},$$

where $h_{\omega }=\omega \rho_{b}c_{b}⩾0$. The domain (Ω) is spatially discretized into *M* slices of equal thickness $\delta_{r}=\left(a_{2}-a_{1}\right)/M$ (see Figure 5).

Using explicit discretization schemes centered on the node $r_{m}=a_{1}+m\delta_{r}$, the discrete version of (13) can be written as follows:

(14) $$\begin{array}{cc} \rho c\delta_{r}\frac{T_{m}^{n+1}-T_{m}^{n}}{\Delta t_{n}} & =\frac{k}{\delta_{r}}\left(T_{m-1}^{n}-T_{m}^{n}\right)-\frac{k}{\delta_{r}}\left(T_{m}^{n}-T_{m+1}^{n}\right)-\frac{k}{r_{m}}\left(T_{m-1}^{n}-T_{m+1}^{n}\right)\\ \; & -h_{\omega }\left(T_{m}^{n}-T_{0}\right)\delta_{r}+{\dot{q}}_{m}^{n}\delta r, \end{array}$$

where $T_{m}^{n}$ is the absolute temperature of the node $\left(r_{m}\right)$ at time $t_{n}=t_{n-1}+\Delta t_{n}$ with n⩾1; m∈[1,M−1]. Equation (14) can now be rewritten as a thermal energy conservation relation (Godunov conservative form), where the electro-thermal analogies gathered in Table 2 were used:

(15) $$i_{m-\frac{1}{2}}^{\mathrm{th}}+i_{{\dot{q}}_{m}}^{\mathrm{th}}-i_{m+\frac{1}{2}}^{\mathrm{th}}-i_{m}^{\mathrm{th}}-i_{h_{\omega }m}^{\mathrm{th}}-i_{Cm}^{\mathrm{th}}=0.$$

**Figure 6:**
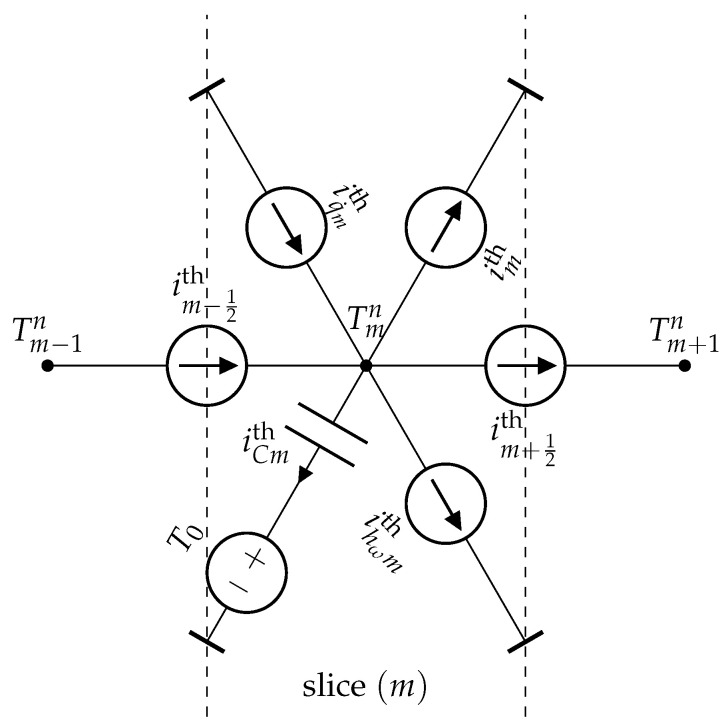
Electro-thermal modeling of a slice (m) of a material obeying the partial differential Equation (12).

Thus, it is possible to give an electro-thermal representation of the slice (m), which is composed of five ideal controlled current sources and one capacitor (see Figure 6). Note that the additional ideal voltage source guarantees an initial temperature of node $\left(r_{m}\right)$ equal to $T_{0}$. Moreover, the capacitance $C^{\mathrm{th}}$ of the analog electro-thermal capacitor is given by $C^{\mathrm{th}}=\rho c\delta_{r}$.

**Figure 7:**
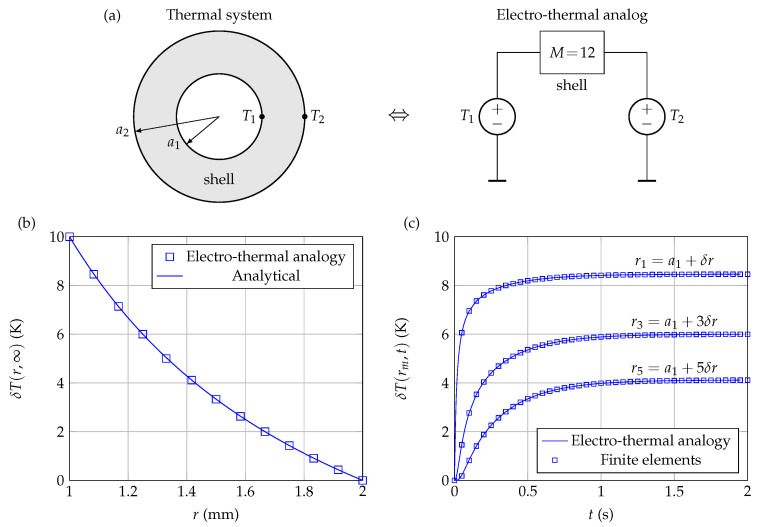
(**a**) Electro-thermal modeling of a shell subjected to Dirichlet’s boundary conditions, without heat volumetric source. (**b**) Stationary temperature profile through the shell. (**c**) Temperature variations as a function of time at different points throughout the shell.

To test the relevance of the Godunov electro-thermal analogy proposed in this work, we first solved Equation (12) in the case where $\dot{q}=0$ and $h_{\omega }=0$, considering the following initial and boundary conditions: $\delta T\left(r,0\right)=T\left(r,0\right)-T_{0}=0$, $\delta T_{2}=T\left(a_{2},t\right)-T_{0}=0$, and $\delta T_{1}=T\left(a_{1},t\right)-T_{0}=10\;K$ (Dirichlet conditions). The considered system is a spherical shell of inner radius $a_{1}=10^{-3}\;m$ and outer radius $a_{2}=2\times 10^{-3}\;m$, with the following physical properties: $k=1.0\;W\cdot m^{-1}\cdot K^{-1}$; $\rho =1.9\times 10^{3}\;\mathrm{kg}\cdot m^{-3}$ and $c=1.5\times 10^{3}\;J\cdot {\mathrm{kg}}^{-1}\cdot K^{-1}$. The spherical shell is discretized into M=12 slices of equal thickness (see Figure 7a). Figure 7b shows the results obtained with the present systemic modeling (symbols) when t→∞ (steady state), for which case there is an analytical solution, given by:

(16) $$\delta T\left(r\right)=T\left(r\right)-T_{0}=\frac{a_{1}\left(a_{2}-r\right)\delta T_{1}+a_{2}\left(r-a_{1}\right)\delta T_{2}}{\left(a_{2}-a_{1}\right)r},$$

It can be seen from Figure 7b that the numerical results are in very good agreement with the analytical results (solid line).

Figure 7c shows the variations of temperature $\delta T(r_{m},t)$ as a function of time at different points $r_{m}$ throughout the shell. The results obtained with the present modeling match remarkably well with those provided by a numerical resolution using the finite elements method (FlexPDE 6.0).

The case of a constant heat volumetric source $\dot{q}=\mathrm{cste}$ was also computed, with the other properties being identical to those used previously. There is still an analytical steady-state solution in this case, and, considering the same Dirichlet boundary conditions as used previously, one gets:

(17) $$\delta T\left(r\right)=\frac{\left(r-a_{2}\right)\left(6a_{1}k\delta T_{1}+a_{1}^{3}\dot{q}\right)+\dot{q}\left[a_{1}\left(a_{2}^{3}-r^{3}\right)+a_{2}r\left(r^{2}-a_{2}^{2}\right)\right]}{6\left(a_{1}-a_{2}\right)kr}.$$

**Figure 8:**
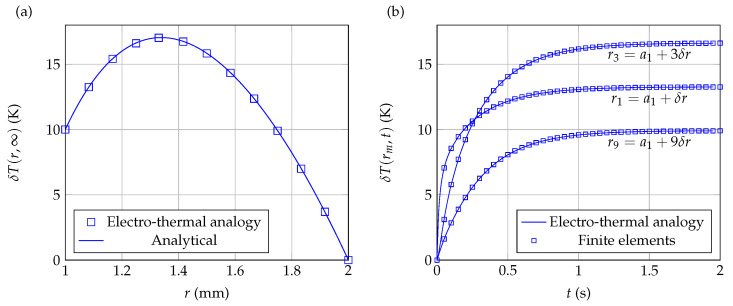
Electro-thermal modeling of a shell subjected to Dirichlet boundary conditions, with a constant heat volumetric source. (**a**) Steady state temperature profile through the shell. (**b**) Temperature variations as a function of time at different points throughout the shell.

Figure 8a (steady state profile) and Figure 8b (temporal variations at different points) show the results obtained with the present approach for $\dot{q}=10^{8}\;W\cdot m^{-3}$ and M=12. Figure 8a compares the results obtained with the present electro-thermal analogy to the analytical solution (17) for the case of steady state profile, while Figure 8b compares the results of the electro-thermal analogy to those obtained by a finite elements solver. In both cases, it can be seen that the correspondence between the two approaches is excellent.

**Figure 9:**
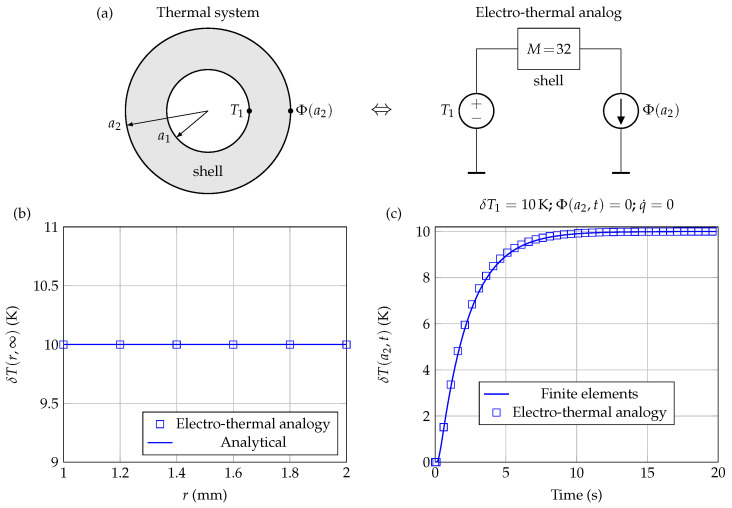
(**a**) Electro-thermal modeling of a shell subjected to Dirichlet boundary condition ($\delta T_{1}=10\;K$) at $r=a_{1}$ and homogeneous Neumann condition at $r=a_{2}$: $\Phi \left(a_{2}\right)={\left(\partial_{r}T\right)}_{r=a_{2}}=0$. (**b**) Steady state temperature profile through the shell. (**c**) Temperature variations as a function of time at $r=a_{2}$.

Using current sources at the boundaries rather than voltage sources (see Figure 9a), it is also possible to simulate Neumann-type boundary conditions: $\Phi \left(a_{i}\right)={\left(\partial_{r}T\right)}_{r=a_{i}}$. In the case of a Dirichlet boundary condition at $r=a_{1}$ and a Neumann boundary condition at $r=a_{2}$, there is an analytical expression of the steady state solution, which is given here by:

(18) $$\delta T\left(r\right)=\frac{\dot{q}\left[r\left(a_{1}^{3}+2a_{2}^{3}\right)-a_{1}\left(2a_{2}^{3}+r^{3}\right)\right]+6ka_{1}r\delta T_{1}+6\Phi \left(a_{2}\right)ka_{2}^{2}\left(r-a_{1}\right)}{6a_{1}kr}.$$

The numerical resolution being more demanding with this kind of boundary condition, it is generally preferable to use a more precise discretization in this case. Figure 9b (steady state profile) and Figure 9c (temporal variations at $r=a_{2}$) compare the results obtained for $\dot{q}=\Phi \left(a_{2}\right)=0$ using the present approach with M=32, to those obtained by a finite elements solver, with the other properties being identical to those used previously. In both cases, it can be seen that the correspondence between the two approaches is still very good.

Finally, solving the bioheat Equation (13) is also considered, with $T_{0}=0\;K$ and using Dirichlet boundary conditions: $T\left(a_{2},t\right)=T_{2}=0\;K$ and $T\left(a_{1},t\right)=T_{1}=1\;K$. Usual values of the physical parameters were chosen from Bergman et al. [38] for human tissues and blood: $\dot{q}=700\;W\cdot m^{-3},$ $h_{\omega }=1800\;W\cdot m^{-3}\cdot K^{-1},$$\rho =1000\;\mathrm{kg}/m^{3}$, $c=3600\;J\cdot {\mathrm{kg}}^{-1}\cdot K^{-1}$, and $k=0.5\;W\cdot K^{-1}\cdot m^{-1}$. Note that an analytical expression also exists here for the steady-state temperature, which is given by:

(19) $$\begin{array}{cc} \begin{array}{cc} T(r,\infty ) & = \end{array}\; & \frac{e^{-\beta r}}{\left(e^{2\beta a_{1}}-e^{2\beta a_{2}}\right)h_{\omega }r}\times \left[\left(e^{\beta \left(2a_{1}+r\right)}-e^{\beta \left(2a_{2}+r\right)}\right)\dot{q}r\right.\\ + & a_{1}\left(e^{\beta \left(a_{1}+2r\right)}-e^{\beta \left(a_{1}+2a_{2}\right)}\right)\left(h_{\omega }T_{1}-\dot{q}\right)\\ + & \left.a_{2}\left(e^{\beta \left(2a_{1}+a_{2}\right)}-e^{\beta \left(2r+a_{2}\right)}\right)\left(h_{\omega }T_{2}-\dot{q}\right)\right], \end{array}$$

where $\beta =\sqrt{h_{\omega }/k}$.

**Figure 10:**
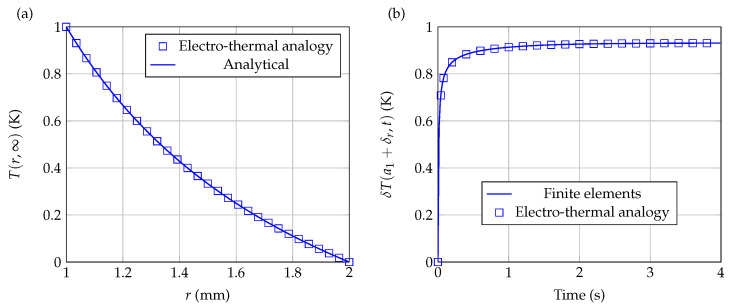
Electro-thermal modeling of bioheat transfer through a shell subjected to Dirichlet boundary conditions and constant heat volumetric source. (**a**) Steady state temperature profile through the shell. (**b**) Temperature variations as a function of time at $r=a_{1}+\delta_{r}$.

Figure 10a compares the results obtained by the present electro-thermal analogy with M=28, to the analytical solution (19) for the case of steady-state profile. The maximum relative error between numerical and analytical solutions is in this case less than $6\times 10^{-4}$. On the other hand, Figure 10b compares the variations of $T(a_{1}+\delta_{r},t)$ as a function of time, given by the electro-thermal analogy, to the variations obtained by a finite elements solver. In both cases, it can be seen that the correspondence between the two approaches is still excellent.

### 3.2. NTC Bead-Type 1D Systemic Modeling

The previous examples have shown that the approach developed in this work can handle a wide variety of spherically symmetric heat transfer problems, close to the problem of thermal characterization of materials using a bead-type NTC. Thus, we are now ready to propose a 1D systemic modeling of a bead-type NTC, immersed in a fluid at rest.

#### 3.2.1. Ideal NTC Bead-Type 1D Systemic Modeling

We first consider the ideal case for which the thermal contact between the active core and the sheath is perfect, and the thermal losses along the connecting copper wires are not taken into account. In order to be consistent with the electro-thermal current densities gathered in Table 2, the energy balance (5) of the active core is divided by the effective surface area of the equivalent spherical core $S_{c}=4\pi a_{c}^{2}$:

(20) $$\left(5\right)/S_{c}\Rightarrow \frac{a_{c}}{3}\rho_{c}c_{c}\frac{dT_{c}}{dt}=\frac{a_{c}}{3}{\dot{q}}_{e}+k_{s}{\left(\frac{\partial T_{s}}{\partial r}\right)}_{r=a_{c}}.$$

**Figure 11:**
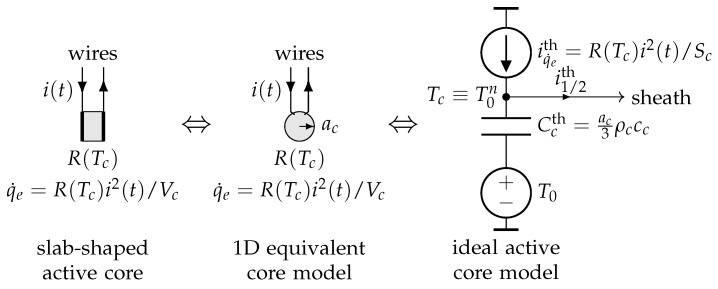
One-dimensional electro-thermal systemic modeling of an ideal NTC active core with electrical resistance $R(T_{c})$ given by Equation (1) or (2). Recall that the effective radius $a_{c}$ is chosen so that the volume $V_{c}$ of the equivalent spherical core is equal to that of the slab.

In the case of an ideal thermal contact between the active core and the sheath, the second term of the right-hand side of (20) is already taken into account in the discretization of the sheath (see Figure 6, node m=1). In this case, $T_{c}\left(t_{n}\right)\equiv T_{0}^{n}$ and the electro-thermal circuit shown in Figure 11 can be used to describe the temporal evolution of the ideal active core uniform temperature $T_{c}\left(t\right)$.

We have found that simulating thermal characterization of materials by self-heating mode, using the ideal NTC core equivalent model shown in Figure 11 led to a systematic and significant overestimation of the thermistor active core temperature rise. This overestimation is due to the fact that the ideal model does not take into account thermal losses along the copper wires which connect the active core at temperature $T_{c}$ to the measurement and control circuit at working temperature $T_{0}<T_{c}$. In practice, these thermal losses limit considerably the temperature rise of the thermistor core, so they must be taken into account if an acceptable systemic modeling of the NTC is to be obtained.

#### 3.2.2. Realistic NTC Bead-Type 1D Systemic Modeling

The ideal model shown in Figure 11 has been corrected by introducing an additional thermal path going from the thermistor core to the measurement circuit. This thermal path has been modeled here by the mean of a thermal resistance $R_{L}$, whose value has been adjusted using experimental results obtained from the thermal characterization of pure liquid glycerol at rest (see Section 4.2). The NTC active core and its insulating shell being both solid bodies, the thermal contact between these two components cannot be strictly ideal. So, a thermal contact resistance $R_{c}$ between the core and the sheath has, therefore, also been introduced into the model. The value of $R_{c}$, which was found to be much less critical than the value of $R_{L}$, was also deduced from the thermal characterization of pure glycerol.

**Figure 12:**
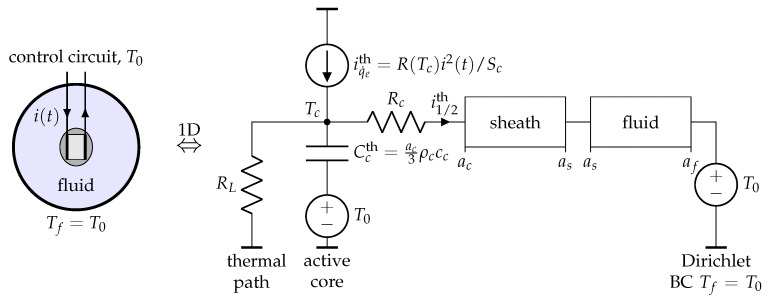
One-dimensional electro-thermal systemic modeling of a bead-type 44004 NTC immersed into a liquid at rest.

Finally, the complete 1D systemic model proposed in this work is represented in Figure 12. It takes into account the following physical properties and phenomena: an uniform temperature of the active core, heat transfers between the core and the sheath and between the sheath and the fluid to be characterized, the spatial discretization of the insulating sheath and of the fluid (in spherical symmetry), thermal paths due to the connecting wires, the contact thermal resistance between the sheath and the active core, and interactions between the NTC and electrical control circuits (through the electrical current i(t) and the electrical resistance $R(T_{c})$).

In order to adequately model the experimental conditions, Dirichlet boundary condition (BC) $T_{f}=T_{0}$ was imposed on the fluid at $r=a_{f}$. The sheath and the fluid elements of Figure 12 were both discretized using the electro-thermal model of Figure 6 and the electro-thermal current densities of Table 2.

Note that it would also be possible to propose a more elementary 1D model than the one developed here. In this more basic model, the active core would be assumed to be punctual, and the heat transfer through the sheath would be modeled using a simple thermal resistance $R_{s}$. The other elements of this point model would be similar to those used in the present 1D model.

## 4. Results

In this last part, experimental results obtained with different 44004 type NTCs are compared to the numerical results provided by the 1D systemic modeling developed in the previous paragraphs.

Glycerol has been chosen here as a reference liquid in order to determine the values of the different effective parameters needed by the 1D NTC spherical equivalent model.

### 4.1. Presentation

As mentioned in Section 2.1, high dynamic viscosity ($\eta_{f}=1.48\;\mathrm{Pa}\cdot s$ at $20{\;}^{\circ }C$ [34]) and thermal conductivity close to that of most biological fluids ($k_{f}=0.285\;W\cdot m^{-1}\cdot K^{-1}$ at $20{\;}^{\circ }C$ [34,39]) made liquid glycerol a good candidate to set the values of the physical parameters entering the 1D systemic modeling of a self-heated NTC used in thermal characterization mode. We recall that a thin layer of insulating varnish has been applied to the NTCs copper connecting wires.

Since NTC manufacturers give very little information about the physical properties of the materials used to produce their thermistors, typical starting values were assigned to a number of unknown parameters used in the model. These values were further refined by minimizing the following quantity, over a large number of tests $\left(N_{\mathrm{test}}⩾20\right)$:

(21) $$\delta T_{c}^{\mathrm{rms}}=\sqrt{\frac{1}{N_{f}}\sum_{n=1}^{N_{f}}{\left(T_{c,n}^{\mathrm{com}}-T_{c,n}^{\mathrm{mea}}\right)}^{2}},$$

where: $T_{c,n}^{\mathrm{com}}$ and $T_{c,n}^{\mathrm{mea}}$ are the computed and measured active core temperatures, respectively, at time $t_{n}=t_{n-1}+\delta t_{n}$; $N_{f}$ is the number of experimental samples recorded during one test.

The model parameters values leading to the minimization of $\delta T_{c}^{\mathrm{rms}}$ for the entire set of tests performed are gathered in Table 3.

Note that:

- the model does not require the knowledge of the active core thermal conductivity $k_{c}$ because the core temperature $T_{c}$ is assumed to be uniform ($k_{c}\gg k_{s}$);
- the values of $k_{s}$ and $\rho_{s}c_{s}$ shown in Table 3 are compatible with usual epoxy values; and
- the value of the shell external radius $a_{s}$ was determined using a caliper. The NTC being a prolate spheroid, an average value was considered.

### 4.2. Glycerol Thermal Characterization

A number of tests have been carried out with glycerol, which have led to the values shown in Table 3. The tests presented in this paper concern the immersion length of the NTC copper connecting wires.

#### 4.2.1. Immersion Length Tests

**Figure 13:**
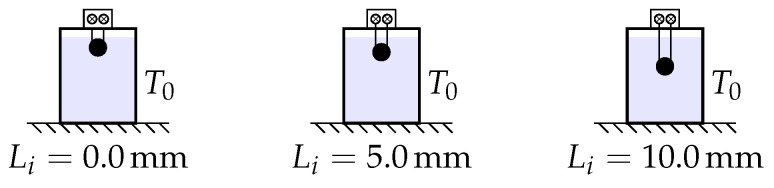
Different immersion lengths $L_{i}$ in glycerol.

In order to test the relevance of the loss resistance $R_{L}$, which is a key parameter of the present systemic model, we have studied the temporal variations of the active core temperature $T_{c}\left(t\right)$ considering four different immersion lengths $L_{i}$ of the same thermistor in the same liquid (glycerol) at rest: $L_{i}=0.0,\;0.1,\;5.0$ and 10.0mm (see Figure 13). The immersion of the NTC in the liquid was carried out using the mobile arm of a digital Vernier caliper providing a precision to 0.01 mm. A miniature digital microscope (with magnification ×10 to ×300) was used to accurately determine the position of the top of the NTC relative to the free surface of the liquid being characterized. The control circuit is that of Figure 4, using the following values: $R_{0}=1497.0\;\Omega$, $T_{0}=298.7\;K$, and different constant excitation voltages $v_{0}$.

**Figure 14:**
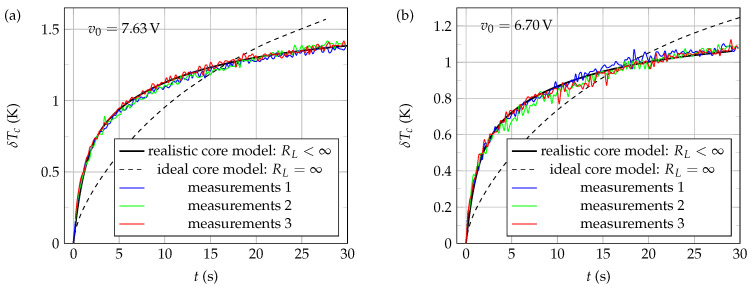
Evolution of the active core temperature $\delta T_{c}\left(t\right)=T_{c}\left(t\right)-T_{0}$ as a function of time *t* when the self-heated thermistor NTC1 is immersed with zero immersion length ($L_{i}=0.0$ mm) in pure glycerol at rest, considering a working temperature $T_{0}=25.0{\;}^{\circ }C$ and constant excitation voltages (**a**) $v_{0}=7.63\;V$ and (**b**) $v_{0}=6.70\;V$. Measurements were conducted using the control circuit of Figure 4.

Three thermistors from the same lot were tested, namely: NTC1, NTC2, and NTC3. Figure 14a,b show typical experimental results (red, green, and blue curves) and numerical results (solid and dashed black curves) obtained when NTC1 is immersed with zero immersion length ($L_{i}=0.0$ mm) in pure glycerol at rest, using two different constant excitation voltages, $v_{0}=7.63\;V$ and $v_{0}=6.70\;V$, respectively. A working temperature $T_{0}=25.0{\;}^{\circ }C$ was considered here. As shown by the dashed black curves, the ideal model (see Figure 11, where $R_{L}=\infty$) does not allow for adequate description of the self-heating of the active core (measurements curves 1 to 3), regardless of the excitation voltage used. In contrast, the introduction of a finite loss resistor in the core model (see Figure 12, where $R_{L}<\infty$) has systematically allowed a very adequate and realistic description of the thermistor active core self-heating, as shown by the black solid curves, which well describe the experimental evolutions of the temperature $T_{c}\left(t\right)$.

Thus, it can be seen from the curves of Figure 14 that introducing a finite loss resistance $R_{L}$ in the 1D systemic model of the NTC is crucial to properly describe the evolution of the core temperature as a function of time.

Note that very similar experimental and numerical results were also found at other operating temperatures (ranging from $20.0{\;}^{\circ }C$ to $40.0{\;}^{\circ }C$), other excitation voltages (ranging from 5.50 V to 8.50 V), and other immersion lengths ($L_{i}=0.1,\;5.0$ and 10.0 mm), for each of the three NTCs tested in this study.

**Figure 15:**
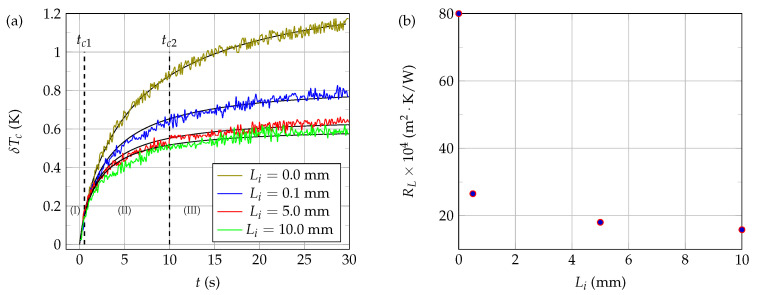
Influence of the immersion length $L_{i}$ on the self-heating of NTC3 immersed in glycerol at rest, at a working temperature $T_{0}=25.0{\;}^{\circ }C$. A constant excitation voltage $v_{0}=6.90$ V was used to heat the NTC. (**a**) Active core temperature evolution as a function of time and length $L_{i}$. The solid black curves correspond to the results given by the 1D electro-thermal systemic modeling while the colored curves correspond to the experimental data. (**b**) Evolution of the loss resistance $R_{L}$ as a function of the immersion length $L_{i}$.

Figure 15a shows the influence of the immersion length $L_{i}$ (and, thus, of the loss resistance $R_{L}$) on the self-heating of the thermistor NTC3 when immersed in glycerol at rest, at the working temperature $T_{0}=25.0{\;}^{\circ }C$. The values of the loss resistance corresponding to each length $L_{i}$ (see Figure 15b) and the value of the contact resistance $R_{c}$ (see Table 3) were determined by minimization of (21), using the same number of samples $N_{f}=300$ for each set of measurements. From the modeling point of view, both the equivalent insulating sheath and the fluid domain were discretized with the numerical scheme exposed in Section 3.1, using the same number *M* of layers (M=28) in both cases. The radius $a_{f}$ of the fluid domain was chosen equal to that of the measurement cell: $a_{f}=4.5$ mm. The values of the other parameters required for the numerical modeling (except $R_{L}$) were those indicated in Table 3.

As it can be seen from the curves shown in Figure 15a, the self-heating of the thermistor core is greater the shorter the immersion length, all other factors being equal. This clearly indicates that there are heat exchanges between the connecting wires and the liquid to be characterized and that these exchanges are greater the longer the length of wire immersed, as expected. However, the non-linearity of the variations of $R_{L}$ with $L_{i}$ (see Figure 15b) reflects the great complexity of the thermal exchanges between the connecting wires and the fluid to be characterized. Moreover, the fact that $R_{L}$ is not infinite when $L_{i}=0.0$ mm also reflects the existence of thermal transfers between the NTC core and the connector system which connects the electrical control circuit to the NTC copper wires, which are acting as thermal paths.

The heat sink property of the connecting wires is also well illustrated by the results gathered in Table 4. When $L_{i}>0.0\;\mathrm{mm}$, it can be seen from this table that the thermal power current $i_{L}^{\mathrm{th}}$, which is evacuated via the connection wires, represents more than 50% of the total thermal power $i_{{\dot{q}}_{e}}^{\mathrm{th}}$ supplied by the control circuit to the NTC at $t=t_{f}$ and is, therefore, not used for the thermal characterization of the fluid. However, it can also be observed from this table that the thermal power fraction $i_{C}^{\mathrm{th}}$, that only serves to raise the active core temperature, has become negligible at $t=t_{f}$, whatever the value of $L_{i}$. Meanwhile, the fraction $i_{S+F+T_{0}}^{\mathrm{th}}$ of the thermal power that passes through the sheath, then the fluid, and, finally, reaches the thermostat is considerable and allows, despite the losses, for adequately probing the fluid to be characterized, as well as for the four $L_{i}$ values considered in this study.

Note that the low values of $\delta T_{c}^{\mathrm{rms}}$ gathered in Table 4 confirm the relevance of the modeling proposed in this paper.

In conclusion, we found in this study that the value of the loss resistance $R_{L}$ considered in the systemic model was highly dependent on the immersion length $L_{i}$. This dependence has a considerable influence on the results provided by the modeling and, thus, on the good correspondence between numerical and experimental results (see Figure 14a,b, for example). In the context of the present approach, therefore, it is essential to access to the value of the loss resistance with a high degree of accuracy. Unfortunately, the value of the loss resistance corresponding to a given immersion length $L_{i}\neq 0$ depends on the nature of the considered liquid to be characterized. Therefore, when $L_{i}\neq 0$, it is impossible to determine both $R_{L}$ and another unknown characteristic of the fluid (such as thermal conductivity, for example). Therefore, it is generally recommended, on the one hand, to limit as much as possible thermal exchanges between the connection wires and the liquid to be characterized (for example, by limiting the immersion length of the connecting wires or by highly isolating them) and, on the other hand, to ensure a very precise and reproducible positioning of the NTC (because of the high value of the slope of $R_{L}\left(L_{i}\right)$ when $L_{i}\to 0$).

Among the four immersion lengths considered in this study, we noted that the length $L_{i}=0.0$ mm was the most relevant, which is consistent with the previous discussion. Indeed, it can be seen from Table 4 that this specific length allows, on the one hand, for obtaining the lowest thermal losses; on the other hand, it leads to the highest fluid probing thermal current $i_{S+F+T_{0}}^{\mathrm{th}}$, which is in favor of good thermal characterizations. Moreover, the heat exchanges between the fluid to be characterized and the connecting wires are negligible in the case where $L_{i}=0.0\;\mathrm{mm}$. Therefore, the value of the loss resistance becomes fluid independent in this particular case, which is a great advantage for thermal characterization, as mentioned previously.

#### 4.2.2. Thermal Power Balance and Characteristics Times

The 1D systemic modeling introduced in this paper also allows access to quantities that are difficult or impossible to measure, such as the various thermal current densities that flow through the system during thermal characterization.

**Figure 16:**
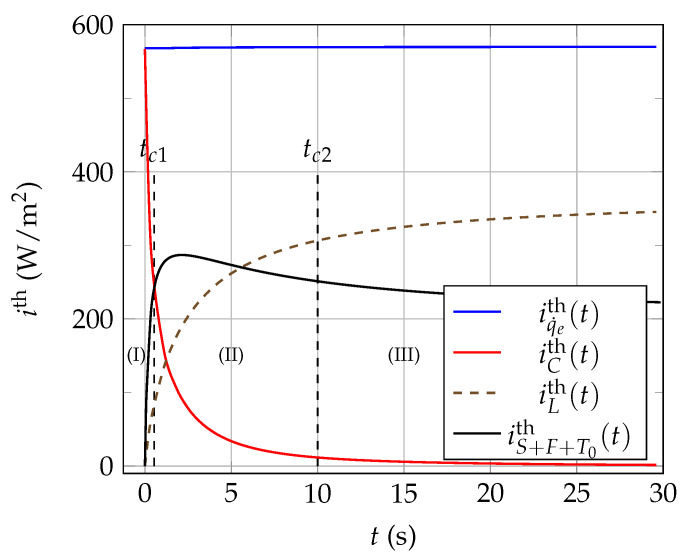
Evolution of the different thermal current densities as a function of time t∈[0,30] s, calculated at $r=a_{c}$, in the case of NTC3 immersed in pure glycerol at rest, when: $L_{i}=5.0\;\mathrm{mm}$, $v_{0}=6.90\;V$, and $T_{0}=25{\;}^{\circ }C$.

The analysis of the temporal variations of the thermal powers exchanged during the thermal characterization of glycerol provides useful information about the operation of a NTC in thermal characterization mode. Figure 16 shows the evolution of the thermal power current densities (in $W/m^{2}$) calculated at position $r=a_{c}$ as a function of time t∈[0,30] s.

Analyzing the curves shown in Figure 16 reveals the existence of mainly three different temporal domains, delimited by two characteristics times $t_{c1}$ and $t_{c2}$ for which we get $i_{C}^{\mathrm{th}}\left(t_{c1}\right)\approx i_{S+F+T_{0}}^{\mathrm{th}}\left(t_{c1}\right)$ and $i_{C}^{\mathrm{th}}\left(t_{c2}\right)\ll i_{S+F+T_{0}}^{\mathrm{th}}\left(t_{c2}\right)$, respectively. The operation of the NTC on each of these three temporal domains can be described as follows:

- when $0⩽t<t_{c1}$, time domain (I): only the thermal current density $i_{C}^{\mathrm{th}}$ is significant, and the thermal power supplied by the electrical control circuit mainly serves here to raise the temperature $T_{c}$ of the NTC active core. This time domain cannot be used to characterize the fluid surrounding the NTC because the evolution of $T_{c}\left(t\right)$ between 0 and $t_{c1}$ is mainly influenced here by the physical properties of the active core and not by the physical properties of the surrounding fluid to be characterized, that is not yet probed by the thermal waves emitted by the NTC. In addition, we can see in Figure 15a that the curves giving the experimental temporal evolution of $T_{c}$ coincide with $t\in [0,t_{c1})$ and this, whatever the value of $L_{i}$ and, thus, of $R_{L}$. This observation confirms that the evolution of $T_{c}\left(t\right)$ over the interval $\left[0,t_{c1}\right)$ mainly reflects the physical properties of the thermistor active core only.
  Therefore, it is this particular time domain that was used in the present systemic modeling to determine the optimal values of the effective $a_{c}$ and $\rho_{c}c_{c}$ parameters, by minimizing (21) on $\left[0,t_{c1}\right)$ (see Table 3).
- When $t_{c1}<t<t_{c2}$, time domain (II): in the case of Figure 16, the three thermal current densities $i_{C}^{\mathrm{th}}$, $i_{L}^{\mathrm{th}}$, and $i_{S+F+T_{0}}^{\mathrm{th}}$ have comparable values; therefore, the thermal characterization of the surrounding fluid using the time evolution of the core temperature will be influenced by both the properties of the core, of the insulating shell and by the thermal losses via the connecting wires. Given these various influences, the usual transient methods of thermal characterization (which do not use systemic modeling) based on bead-type NTCs should avoid the use of domain (II) data.
- When $t>t_{c2}$, time domain (III): in this case, the thermal current density $i_{C}^{\mathrm{th}}$ has become negligible in front of $i_{L}^{\mathrm{th}}$ and $i_{S+F+T_{0}}^{\mathrm{th}}$, and it can be supposed that the properties of the core are then without any significant influence on the temporal evolution of its temperature $T_{c}$. Consequently, it is this time domain that should be used preferably for thermal characterization of materials by using bead-type NTCs self-heating methods. However, since thermal losses and the insulating sheath still have an influence on the temporal evolution of $T_{c}$ in the (III) domain, thermal characterization methods that do not use systemic modeling must imperatively resort to a prior calibration of the measuremen device by using several reference fluids, such as glycerol, ethanol, water–glycerol mixtures, and gelled water (using agar-agar, for example).
  Note that, in this work, the characteristic time $t_{c2}$ has been calculated using the following relation: $i_{C}^{\mathrm{th}}\left(t_{c2}\right)\approx 3\cdot 10^{-2}\times i_{C}^{\mathrm{th},\max}$, where $i_{C}^{\mathrm{th},\max}=i_{C}^{\mathrm{th}}\left(0_{+}\right)$.

The 1D systemic modeling proposed in this work allowed to highlight the existence of two characteristic times of the thermal transfers, when bead-type NTCs are used for thermal characterization of materials by self-heating methods. The knowledge of these characteristic times can help to a better understanding of the operation of bead-type NTCs used for thermal characterization of materials, as it will be illustrated in the following paragraph.

#### 4.2.3. Constant Voltage Excitation Signal Processing

Various kinds of possible electrical excitations can be consider to heat the NTCs used in transient thermal characterization methods: current step through the NTC (using a precise current source) and resistance step (used in constant temperature heating techniques (CTHT)), and voltage step (using a divider bridge). From the point of view of the control circuit, thermal characterization using a constant voltage excitation is relatively easier to implement than CTHT; thus, it is very common to use a voltage step to heat NTCs in transient thermal characterization. Several signal processing methods can be considered in order to extract the thermal conductivity from the temporal variations of $T_{c}\left(t\right)$ in the case of a voltage step excitation.

For example, Kharalkar et al. proposed to write the quantity $P_{e}\left(t\right)/\delta T_{c}\left(t\right)$ in the following form [11]:

(22) $$\frac{P_{e}\left(t\right)}{\delta T_{c}\left(t\right)}=D_{0}+D_{1}t^{-1/2}.$$

They then proposed to express the thermal conductivity $k_{f}$ of the fluid to be characterized as $k_{f}=1/\left(a_{1}D_{0}^{-1}+a_{2}\right)$, where $a_{1}$ and $a_{2}$ are empirical coefficients depending on both the thermistor considered and the thermal losses. These coefficients are determined by a precise calibration, at given voltage excitation, temperature, and immersion length, using at least two reference materials with thermal conductivities close to the one being measured.

**Figure 17:**
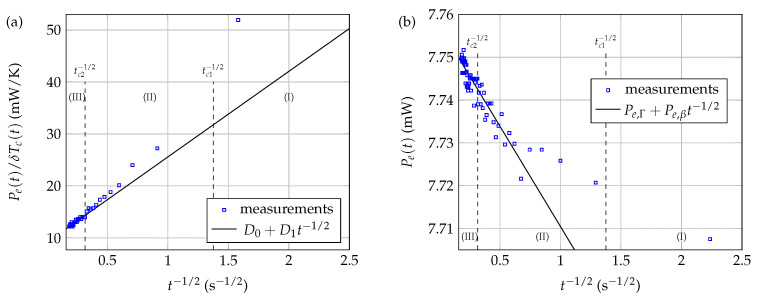
Signal processing of the experimental thermal signals obtained by constant voltage excitation in the case of NTC3 immersed in pure glycerol at rest, when $L_{i}=5.0\;\mathrm{mm}$, $v_{0}=6.90\;V$, and $T_{0}=25{\;}^{\circ }C$: (**a**) Kharalkar et al. signal processing; (**b**) CTHT-type signal processing. Fitting curves (straight lines) were calculated using data belonging only to time domain (III).

In the present paragraph, we consider the processing of the experimental data shown in Figure 15a, obtained when $L_{i}=5.0$ mm. As can be seen from Figure 17a, the model proposed by Kharalkar et al. (solid black line) applies well to the experimental measurements, provided only instants $t>t_{c2}$ are considered (i.e., time domain (III) in Figure 15a, Figure 16 and Figure 17a).

Other signal processing can also be considered. As can be seen from Figure 17b, a modeling in the form of a CTHT type law $P_{e}\left(t\right)=P_{e,\beta }t^{-1/2}+P_{e,\Gamma }$ also provides an acceptable description of the experimental measurements, even in the case of constant voltage heating, provided only data of time domain (III) are considered.

### 4.3. Liquids Thermal Conductivity Measurements

In this paragraph, a new method of thermal conductivity measuring is introduced, which we describe as quasi-absolute, since it requires the use of only one reference liquid.

#### 4.3.1. Electro-Thermal Systemic Modeling (ESM) Method

The present 1D electro-thermal systemic modeling (ESM) approach was applied to the determination of the thermal conductivity $k_{f}$ of three different water/glycerol mixtures, using the following water mass ratios: 100% water (100W0G), 50% water (50W50G), and 40% water (40W60G). Thermistor NTC3 was used in constant voltage heating mode (using the circuit of Figure 4), with $L_{i}=0.0\;\mathrm{mm}$.

The implemented protocol was as follows:

1. Determination of the model parameters values $a_{s}$, $a_{c}$, $\rho_{c}c_{c}$, $\rho_{s}c_{s}$, $k_{s}$, $R_{c}$, and $R_{L}$ from the thermal characterization of pure glycerol at rest, when $L_{i}=0.0$ mm (see Section 4.2).
  The model parameters values obtained from the glycerol thermal characterization (see Table 3 and Table 4) were used in this work for the determination of the thermal conductivities of the three water/glycerol mixtures.
2. Voltage step excitation of the NTC (using the circuit of Figure 4) precisely immersed at $L_{i}=0.0$ mm in the liquid to characterize, at constant working temperature, $T_{0}$. The experimental time variations of the NTC core temperature $T_{c}^{\mathrm{mea}}\left(t\right)$ were extracted from the $v_{0}\left(t\right)$ and $v_{1}\left(t\right)$ voltages, recorded using a data acquisition board (see Section 2.3).
3. Determination of the measured thermal conductivity value $k_{f}^{\mathrm{mea}}$ by minimization of $\delta T_{c}^{\mathrm{rms}}$, given by Equation (21), as a function of the thermal conductivity value $k_{f}$ used in the systemic model. The values of the fluid density $\rho_{f}$ and its specific heat $c_{f}$ are supposed known.

#### 4.3.2. Pure Water Liquid (100W0G)

In the case of pure water (100W0G), a small amount of agar-agar powder has been added to 100W0G samples in order to limit the influence of free convection.

**Figure 18:**
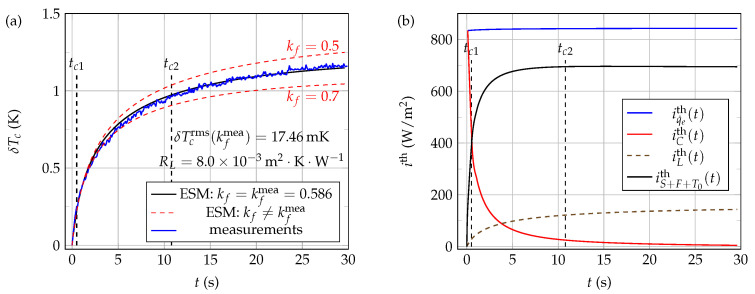
Experimental and 1D ESM computed thermal signals in the case of pure water at rest. These signals were obtained using a voltage step excitation $v_{0}=8.46\;V$ at a working temperature $T_{0}=24.0{\;}^{\circ }C$ and immersion length $L_{i}=0.0\;\mathrm{mm}$: (**a**) measurements and electro-thermal systemic modeling results; (**b**) computed thermal current densities when the fluid thermal conductivity $k_{f}$ is set to the value $k_{f}^{\mathrm{mea}}=0.586$ in the ESM model. Thermal conductivities $k_{f}$ are given in $W\cdot K^{{}^{-1}}\cdot m^{-1}$.

Figure 18a shows typical experimental (blue line) and 1D ESM computed (black and red lines) time evolutions of the NTC active core temperature $\delta T_{c}=T_{c}-T_{0}$, when the thermistor is immersed at $L_{i}=0.0\;\mathrm{mm}$ in pure water at rest, at a working temperature $T_{0}=24.0{\;}^{\circ }C$ and using a voltage step excitation $v_{0}=8.46\;V$. The electrical circuit used to both excite the NTC and record the data is that shown in Figure 4, using the same settings as those chosen for the thermal characterization of glycerol (see Section 4.2.1). The correspondence between the experimental measurements (blue curve) and systemic modeling (black curve) is very good when the value of $k_{f}$ is set in the model to $k_{f}^{\mathrm{mea}}=0.586\;W\cdot K^{{}^{-1}}\cdot m^{-1}$, as reflected by the low value of $\delta T_{c}^{\mathrm{rms}}$ in this case. In addition, note that the very good correspondence between calculated and experimental curves over the time domain $\left[0,t_{c1}\right]$ confirms the relevance of the NTC core effective physical parameters values obtained from the thermal characterization of pure glycerol.

**Figure 19:**
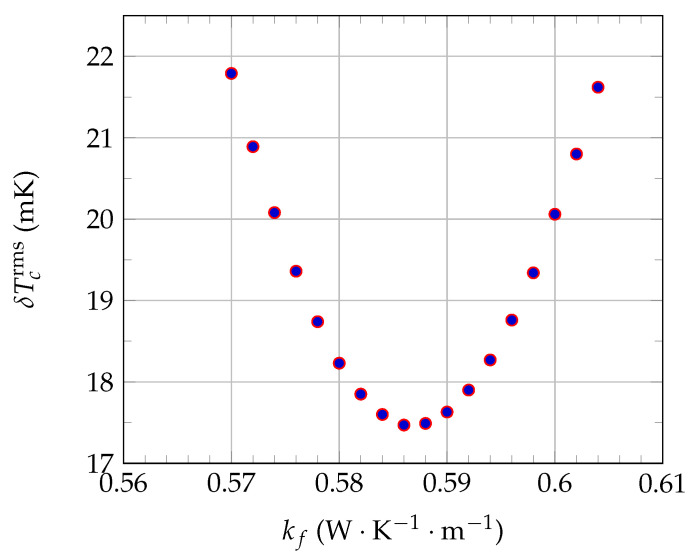
Principle of the determination of pure water thermal conductivity $k_{f}^{\mathrm{mea}}$ by the present 1D electro-thermal systemic modeling (ESM) approach. The values of pure water density $\rho_{f}$ and specific heat $c_{f}$ at $T_{0}=24.0{\;}^{\circ }C$ used in the systemic modeling are given in Table 1.

The red dashed curves shown in Figure 18a illustrate the $\delta T_{c}^{\mathrm{rms}}$ minimization steps. By gradually varying the value of $k_{f}$ used in the systemic model (from $k_{f}=0.5$ to $0.7\;W\cdot K^{-1}\cdot m^{-1}$, for example), all other quantities being held constant, $\delta T_{c}^{\mathrm{rms}}$ is varied accordingly (see Figure 19). Thus, the value of $k_{f}$ that gives the smallest value of $\delta T_{c}^{\mathrm{rms}}$ is the measured value $k_{f}^{\mathrm{mea}}$ of the thermal conductivity of the fluid.

Finally, the curves presented in Figure 18b show in the present configuration (pure liquid water at rest, $v_{0}=8.46\;V$, and $L_{i}=0.0$ mm), when $t>t_{c2}$, that the thermal current density $i_{S+F+T_{0}}^{\mathrm{th}}$ is quite larger here than both $i_{L}^{\mathrm{th}}$ and $i_{C}^{\mathrm{th}}$. Thus, it can be concluded that bead type NTC self-heating methods are in the present configuration quite adequate for thermal characterization of pure water at rest.

#### 4.3.3. Glycerol–Water Mixtures 50W50G and 40W60G

The same measurements as in Section 4.3.2 were reproduced both in the case of 40W60G and 50W50G mixtures, under the same conditions as in the case of pure water. The electrical circuit used to both excite the NTC and record the experimental data was that shown in Figure 4, using the same settings as those chosen for the thermal characterization of pure glycerol at rest.

The values of the glycerol–water mixtures densities $\rho_{f}$ and specific heats $c_{f}$ used in the 1D systemic modeling were taken from the open-source thermophysical properties library CoolProp [40].

##### Glycerol–Water Mixture 40W60G

**Figure 20:**
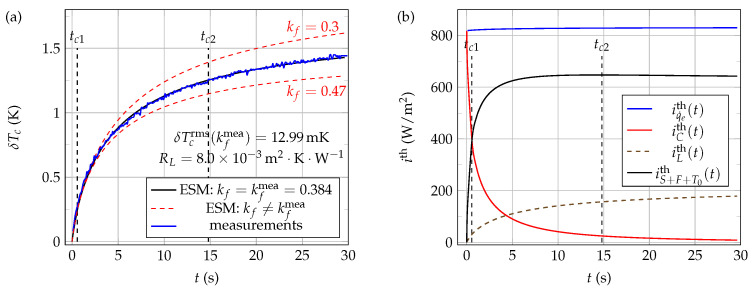
Experimental and 1D ESM computed thermal signals in the case of water–glycerol mixture 40W60G at rest. These signals were obtained using a voltage step excitation $v_{0}=8.39\;V$ at a working temperature $T_{0}=24.0{\;}^{\circ }C$ and immersion length $L_{i}=0.0\;\mathrm{mm}$: (**a**) measurements and ESM results; (**b**) computed thermal current densities when the fluid thermal conductivity $k_{f}$ is set to the value $k_{f}^{\mathrm{mea}}=0.384$ in the ESM model. Thermal conductivities $k_{f}$ are given in $W\cdot K^{{}^{-1}}\cdot m^{-1}$.

Figure 20a shows typical experimental (blue line) and ESM computed (black lines) time evolutions of the NTC active core temperature $\delta T_{c}$, when the thermistor is immersed at $L_{i}=0.0\;\mathrm{mm}$ in 40W60G mixture at rest, at a working temperature $T_{0}=24.0{\;}^{\circ }C$ and using a voltage step excitation $v_{0}=8.39\;V$. The correspondence between the experimental measurements (blue curve) and the electro-thermal systemic modeling (black curve) is very good here when the value of $k_{f}$ is set to $k_{f}^{\mathrm{mea}}=0.384\;W\cdot K^{{}^{-1}}\cdot m^{-1}$ in the model, as reflected by the low value of $\delta T_{c}^{\mathrm{rms}}$ obtained in the present case.

Finally, as in the case of pure water thermal characterization, the curves presented in Figure 20b show that the thermal current density $i_{S+F+T_{0}}^{\mathrm{th}}$ is quite larger here than both $i_{L}^{\mathrm{th}}$ and $i_{C}^{\mathrm{th}}$ when $t>t_{c2}$. Therefore, it can, thus, be concluded that methods based on self-heating of bead-type NTCs are quite adequate for thermal characterization of 40W60G mixtures.

##### Glycerol–Water Mixture 50W50G

**Figure 21:**
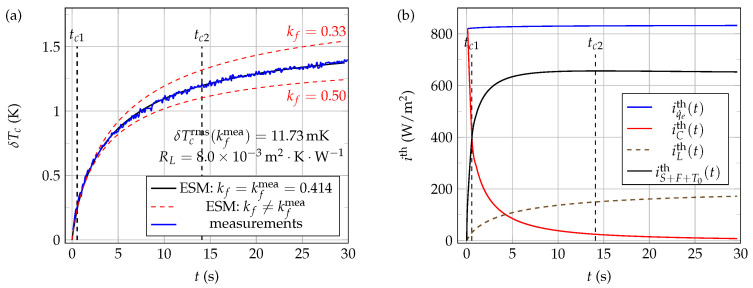
Experimental and 1D ESM computed thermal signals in the case of water–glycerol mixture 50W50G at rest. These signals were obtained using a voltage step excitation $v_{0}=8.39\;V$ at a working temperature $T_{0}=22.0{\;}^{\circ }C$ and immersion length $L_{i}=0.0\;\mathrm{mm}$: (**a**) measurements and ESM results; (**b**) computed thermal current densities when the fluid thermal conductivity $k_{f}$ is set to the value $k_{f}^{\mathrm{mea}}=414$ in the ESM model. Thermal conductivities $k_{f}$ are given in $W\cdot K^{{}^{-1}}\cdot m^{-1}$.

Figure 21a shows typical experimental (blue line) and ESM computed (black lines) time evolutions of the NTC active core temperature $\delta T_{c}$, when the thermistor is immersed at $L_{i}=0.0\;\mathrm{mm}$ in 50W50G mixture at rest, at a working temperature $T_{0}=22.0{\;}^{\circ }C$ and using a voltage step excitation $v_{0}=8.39\;V$. The correspondence between the experimental measurements (blue curve) and the electro-thermal systemic modeling (black curve) is very good here when the value of $k_{f}$ is set to $k_{f}^{\mathrm{mea}}=0.414\;W\cdot K^{{}^{-1}}\cdot m^{-1}$ in the model, as reflected by the low value of $\delta T_{c}^{\mathrm{rms}}$ obtained in the present case.

As in the case of pure water and 40W60G mixture thermal characterizations, the curves presented in Figure 21b show that the thermal current density $i_{S+F+T_{0}}^{\mathrm{th}}$ is again quite larger here than both $i_{L}^{\mathrm{th}}$ and $i_{C}^{\mathrm{th}}$. Therefore, it can still be concluded here that methods based on self-heating of bead-type NTCs are quite adequate for thermal characterization of 50W50G mixtures.

#### 4.3.4. Synthesis

The series of measurements exposed in the previous paragraphs (Section 4.3.2 and Section 4.3.3) were repeated N=12 times for each of the three liquids studied in the present work. A statistical analysis was then applied to these measurements, leading to the results collected in Table 5.

The results gathered in Table 5 show that the 1D ESM approach introduced in this paper gives very suitable results for each of the three liquids that were characterized in the present study. Although this new approach uses only one reference liquid (pure glycerol here), it can be seen from Table 5 that this method allows acceptable measurements of thermal conductivities of liquids with large thermal contrasts (e.g., pure water and 40W60G mixture), as well as liquids with fairly close thermal conductivities (e.g., 40W60G and 50W50G mixtures).

However, it is also observed from Table 5 that the standard error of measurement δk decreases with increasing glycerol content. This decrease is probably due to the fact that it is glycerol that has been used here as the reference liquid. Despite this tendency, it was found in all cases that the measurements were quite accurate. Even in the worst case (pure water here), where the value $\delta k/\bar{k}=2.3\;\%$ was obtained, it was found that the measured value $\bar{k}=0.607\;W\cdot K^{{}^{-1}}\cdot m^{-1}$ was very close to the reference value $k_{\mathrm{ref}}=0.605\;W\cdot K^{{}^{-1}}\cdot m^{-1}$.

## 5. Concluding Remarks and Perspectives

A new method for measuring the thermal conductivity of liquids, with the advantage of requiring only one reference liquid, has been introduced and described in this work. This new quasi-absolute approach has proved to be easy to implement, fast (each series of measurements typically takes less than one minute), and quite accurate, regardless of the thermal conductivity range covered during the measurements.

The development of this new thermal characterization method was made possible through the use of a realistic 1D electro-thermal systemic modeling of the whole system (Fluid/Sensor/Connectics/Control electrical circuit), based on a Godunov-SPICE type discretization scheme. This systemic modeling has allowed to highlight two characteristic times of heat transfers within a self-heated thermistor, allowing for better understanding of the operating of bead-type NTCs when they are used for thermal characterization of liquids at rest.

The discretization scheme and the 1D systemic modeling (including the NTC sensor, the electrical control circuit and the materials to characterize) used in the present study were fully described and documented in the paper. Note that the different computer codes (written in Python 3.8) used for the control of the electrical circuit and the processing of the experimental measurements, as well as the SPICE scripts used for computing the systemic modeling numerical results, are available upon request from the author.

The perspectives of this work are numerous. First, from the point of view of thermal characterization, other viscous reference materials (such as pastes and gels) will be tested, and the method will be applied to the measurement of a wide range of thermal conductivities, as a function of temperature. We believe that the precise positioning of the NTC sensor at the specific immersion length $L_{i}=0.0$ mm can quite easily be automated. This precise and automated positioning of the sensor could allow the present method to access a wide variety of fields, such as the thermal characterization of biological materials (in-vitro and in-vivo) and nanofluids, in the laboratory or in industrial processes. We believe that this approach is also of interest in the case of dynamic processes, such as the drying of complex suspensions, where the thermal conductivity and the level of the free surface of the suspension can change over time.

By changing the boundary condition considered in the present 1D systemic modeling (at the interface between the insulating shell and the fluid), it will also be possible to consider the application of the present approach to the measurement of fluids thermal characteristics when they are flowing.

Systemic 1D modeling of positive temperature coefficient (PTC) silicon thermistors (silistor) is also considered as a possible extension of the present work.

## Figures and Tables

**Table 1:** Typical physical properties values water (at 20 ${}^{\circ }C$ and 24 ${}^{\circ }C$, from reference [35]) at atmospheric pressure. The units of α and β are given in the text.

	$k\;(W\cdot m^{-1}\cdot K^{-1})$	$\rho \;(\mathrm{kg}/m^{3})$	$c\;(\mathrm{kJ}\cdot {\mathrm{kg}}^{-1}\cdot K^{-1})$	η(Pa·s)	α $/10^{-8}$	β $/10^{-4}$
Water (20 ${}^{\circ }C$)	0.598	998.2	4.19	$1.00\times 10^{-3}$	14.3	2.1
Water (24 ${}^{\circ }C$)	0.605	997.3	4.18	$9.11\times 10^{-4}$	14.5	2.5

**Table 2:** Analog electrical and thermal current densities (in $W\cdot m^{-2}$).

Thermal	Electro-Thermal
$\frac{k}{\delta_{r}}\left(T_{m-1}^{n}-T_{m}^{n}\right)$	$i_{m-\frac{1}{2}}^{\mathrm{th}}$
$\frac{k}{\delta_{r}}\left(T_{m}^{n}-T_{m+1}^{n}\right)$	$i_{m+\frac{1}{2}}^{\mathrm{th}}$
$\frac{k}{r_{m}}\left(T_{m-1}^{n}-T_{m+1}^{n}\right)$	$i_{m}^{\mathrm{th}}$
${\dot{q}}_{m}^{n}\delta r$	$i_{{\dot{q}}_{m}}^{\mathrm{th}}$
$h_{\omega }\delta_{r}\left(T_{m}^{n}-T_{0}\right)$	$i_{h_{\omega }m}^{\mathrm{th}}$
$\rho c\delta_{r}\frac{T_{m}^{n+1}-T_{m}^{n}}{\Delta t_{n}}$	$i_{Cm}^{\mathrm{th}}$

**Table 3:** Optimal parameters values deduced from glycerol thermal characterization tests. The units of the two volumetric heat capacities $\rho_{c}c_{c}$ and $\rho_{s}c_{s}$ are given in $J\cdot K^{-1}\cdot m^{-3}$.

$R_{c}\;(m^{2}\cdot K/W)$	$\rho_{s}c_{s}$	$\rho_{c}c_{c}$	$a_{c}\;(\mathrm{mm})$	$a_{s}\;(\mathrm{mm})$	$k_{s}\;(W\cdot K^{-1}\cdot m^{-1})$
$3.0\times 10^{-4}$	$6.72\times 10^{6}$	$3.56\times 10^{6}$	1.04	1.17	0.95

**Table 4:** NTC3 self-heating 1D modeling results, when immersed at $L_{i}$ in glycerol at rest, at $T_{0}=25.0{\;}^{\circ }C$ and $v_{0}=6.90$ V. The thermal power current densities values (in $W\cdot m^{-2}$) were computed at $t_{f}=30\;s$ and $r=a_{c}$. The values of $\delta T_{c}^{\mathrm{rms}}$ were calculated from the results shown in Figure 15a.

$L_{i}$ (mm)	$R_{L}\;(m^{2}\cdot K/W)$	$\delta T_{c}^{\mathrm{rms}}\;(\mathrm{mK})$	$i_{{\dot{q}}_{e}}^{\mathrm{th}}$	$i_{L}^{\mathrm{th}}$	$i_{C}^{\mathrm{th}}$	$i_{S+F+T_{0}}^{\mathrm{th}}$
0.0	$80.0\times 10^{-4}$	18.3	567.2	144.4	7.7	415.1
0.5	$26.5\times 10^{-4}$	19.6	568.5	289.4	2.5	276.6
5.0	$18.0\times 10^{-4}$	18.6	569.9	345.8	1.5	222.6
10.0	$15.8\times 10^{-4}$	24.0	571.7	365.3	1.2	205.2

**Table 5:** Determination of the thermal conductivities of several liquids (pure water and water–glycerol mixtures) by the present 1D ESM approach, with: $\bar{k}$ the mean value of measurements, $\delta k=\sigma_{k}/\sqrt{N-1}$ the standard error of measurement, $\sigma_{k}$ the standard deviation of measurements, and $k_{\mathrm{ref}}$ the reference thermal conductivity value (all these quantities are given in $W\cdot K^{{}^{-1}}\cdot m^{-1}$).

Liquid	$T_{0}$ ${(}^{\circ }C)$	$\bar{k}$	δk	$\delta k/\bar{k}$ (%)	$k_{\mathrm{ref}}$	$\left|k_{\mathrm{ref}}/\bar{k}-1\right|$ (%)
Water (100W0G)	24.0	0.607	0.014	2.3	0.605	0.3
50W50G	22.0	0.4114	0.0049	1.2	0.4189	1.8
40W60G	24.0	0.3905	0.0027	0.7	0.3876	0.7

## Data Availability

The data supporting the reported results were conducted by the author and can be found from him.

## Abbreviations

The following abbreviations are used in this manuscript:

HVAC	Heating, Ventilation, and Air Conditioning
SPICE	Simulation Program with Integrated Circuit Emphasis
THW	Transient Hot Wire
THS	Transient Hot Strip
TPS	Transient Plane Source
NTC	Negative Temperature Coefficient
HMW	Hot Metal Wire
HMF	Hot Metal Film
RTD	Resistance Temperature Detector
ODE	Ordinary Differential Equation
PDE	Partial Differential Equation
ESM	Electro-thermal Systemic Modeling
ADC	Analog to Digital Converter
DAC	Digital to Analog Converter
OA	Operational Amplifier
BC	Boundary Condition
CTHT	Constant Temperature Heating Technique
